# Dendrimer Platforms for Targeted Doxorubicin Delivery—Physicochemical Properties in Context of Biological Responses

**DOI:** 10.3390/ijms25137201

**Published:** 2024-06-29

**Authors:** Magdalena Szota, Urszula Szwedowicz, Nina Rembialkowska, Anna Janicka-Klos, Daniel Doveiko, Yu Chen, Julita Kulbacka, Barbara Jachimska

**Affiliations:** 1 Jerzy Haber Institute of Catalysis and Surface Chemistry Polish Academy of Sciences, 30-239 Cracow, Poland; 2 Department of Molecular and Cellular Biology, Faculty of Pharmacy, Wroclaw Medical University, 50-367 Wroclaw, Poland; 3 Department of Basic Chemistry, Wroclaw Medical University, 50-367 Wroclaw, Poland; anna.janicka-klos@umw.edu.pl; 4 Department of Physics, University of Strathclyde, Glasgow G4 0NG, UK

**Keywords:** PAMAM dendrimers, doxorubicin, dendrimer-doxorubicin interactions, drug delivery systems, DDS

## Abstract

The unique structure of G4.0 PAMAM dendrimers allows a drug to be enclosed in internal spaces or immobilized on the surface. In the conducted research, the conditions for the formation of the active G4.0 PAMAM complex with doxorubicin hydrochloride (DOX) were optimized. The physicochemical properties of the system were monitored using dynamic light scattering (DLS), circular dichroism (CD), and fluorescence spectroscopy. The Quartz Crystal Microbalance with Dissipation Monitoring (QCM-D) method was chosen to determine the preferential conditions for the complex formation. The highest binding efficiency of the drug to the cationic dendrimer was observed under basic conditions when the DOX molecule was deprotonated. The decrease in the zeta potential of the complex confirms that DOX immobilizes through electrostatic interaction with the carrier’s surface amine groups. The binding constants were determined from the fluorescence quenching of the DOX molecule in the presence of G4.0 PAMAM. The two-fold way of binding doxorubicin in the structure of dendrimers was visible in the Isothermal calorimetry (ITC) isotherm. Fluorescence spectra and release curves identified the reversible binding of DOX to the nanocarrier. Among the selected cancer cells, the most promising anticancer activity of the G4.0-DOX complex was observed in A375 malignant melanoma cells. Moreover, the preferred intracellular location of the complexes concerning the free drug was found, which is essential from a therapeutic point of view.

## 1. Introduction

Doxorubicin hydrochloride (DOX) is classified as an anthracycline antibiotic with anticancer properties. Due to its high cytotoxicity, it is widely used as an effective chemotherapeutic agent [1,2]. However, it is also characterized by side effects such as high cardiotoxicity, poor bioavailability, or a tendency to form fibrillar structures under physiological conditions [3,4]. Doxorubicin’s self-aggregation behavior may be responsible for its cytotoxicity, multidrug resistance, and reduced anticancer activity. In addition, the non-spherical fibrillar aggregates of DOX may impede permeation through bilayers such as cell membranes [4,5]. The aggregation of doxorubicin is favored by its amphiphilic nature due to its structure in both the hydrophilic part of daunosamine and the hydrophobic part of anthracycline [5]. Environmental factors such as pH, temperature, ionic strength, or concentration may also contribute to the aggregation of small drug molecules [4,6]. DOX nanocarriers are being developed to improve their stability and reduce toxicity, particularly cardiotoxicity [7,8]. Currently, the most effective doxorubicin delivery systems are based on liposomal nanocarriers. One such formulation is based on pegylated liposomal doxorubicin (Doxil^®^ or Caelyx^®^ according to the European trademark) [9], which was approved by the FDA in 1995, and is widely used in oncology, demonstrating similar or higher efficacy compared to conventional doxorubicin [10]. The second, liposomal doxorubicin (Myocet^®^), was approved in Europe and Canada in 2000, and the FDA granted it “Fast Track” status as a first-line therapy for the treatment of HER2-positive metastatic breast cancer [11]. The liposomal forms of doxorubicin have lower maximum plasma concentrations and longer circulation times than conventional drugs. The circulation time in the bloodstream of free doxorubicin is 0.2 h. Encapsulating it in liposomal formulations extends this time to 2–3 h, while in pegylated liposomal formulations, up to 55 h [12]. Despite its higher therapeutic index and longer circulation time compared to conventional DOX, therapy with pegylated liposomal doxorubicin often causes dose-related toxicities such as mucositis and skin toxicities. Dermal toxicities are not observed with Myocet and are therefore thought to be related to the PEG surface coating. On the other hand, liposomal DOX did not provide long-term stability in the bloodstream [12]. Other challenges associated with liposomal DOX carriers include a lack of efficacy against cancers exhibiting multidrug resistance and accompanying therapy side effects, such as hypersensitivity or peripheral neuropathy [13,14]. Given the limitations of both free doxorubicin and its commercially available nanocarrier formulations, therapies that can improve its stability and minimize side effects are still being sought.

Poly(amidoamine) dendrimers (PAMAMs) are a group of synthetic polymers with a monodisperse nature and nanometer size. They differ from classical linear polymers by their high structural symmetry, the branching of functional groups, and surface polyvalency [15,16]. Expanding the structure with additional layers of functional groups leads to higher generations of dendrimers with larger sizes and numbers of functional groups. Due to their structural diversity, dendrimers are widely used in drug or gene delivery and biosensing [17]. Their hydrophobic interior can promote the encapsulation process of active substances, while their numerous end groups immobilize them on the surface [16,18]. The dendrimers of the 4th generation (G4.0 PAMAM) are one of the most extensively studied in the literature for drug delivery due to their non-toxicity and high drug retention capacity compared to higher generation dendrimers [19,20]. G4.0 PAMAM is a small nanoparticle with a molar mass of 14 kDa and hydrodynamic and gyration radii of R_H_ = 2.45 ± 0.05 nm and R_q_ = 1.87 ± 0.02 nm, respectively [21]. Because of its properties, the G4.0 PAMAM dendrimer can bind ligands efficiently and diffuse through the brain parenchyma, thereby crossing the blood–brain barrier (BBB) [20,22]. The contribution of dendrimers in nanoformulations undergoing clinical trials highlights their potential in the pharmaceutical industry. The current pioneer in the pharmaceutical market is Starpharma company, which uses its patented dendrimer-based technology in two different ways: as stand-alone therapeutics (SPL7013/VivaGel^®^) or through the use of a dendrimer scaffold (Dendrimer Enhanced Product, DEP^®^) for drug delivery [23]. DEP^®^ with covalently attached drug molecules are designed to improve the activity of anticancer drugs such as docetaxel, cabazitaxel, or irinotecan. These DEP^®^-drug conjugates are in phase II clinical trials [24]. Other examples of dendrimer-based nanodrugs include an anticancer drug conjugate with a PEGylated poly(lysine) dendrimer for the treatment of hematologic and solid tumors (phase I clinical trials) or the G4.0 PAMAM dendrimer N-acetyl-cysteine for the treatment of the COVID-19 disease (phase II clinical trials) [25].

From the application point of view, it is worth highlighting the importance of controlling significant drug parameters such as the concentration and pH in the potential self-aggregation process of doxorubicin when designing nanosystems. The encapsulation of doxorubicin in nanoparticles could improve the chemical stability of doxorubicin and prevent its degradation. Yoncheva et al. showed that the encapsulation of DOX in chitosan–alginate nanoparticles increased its stability and prevented degradation during exposure to light [26]. A similar phenomenon was observed by Bandak et al. for doxorubicin encapsulation in liposomes, which reduced its photodegradation compared to the free drug [27]. Encapsulation in dendrimer nanoparticles can also prevent the degradation of unstable, pH- or light-sensitive drugs [28].

The novelty of the present study is to investigate the effect of doxorubicin deprotonation under alkaline conditions on the interactions with the G4.0 PAMAM dendrimer and verify the performance of the designed nanosystem in vitro. UV-vis spectroscopy monitored the stability and partial degradation of DOX under basic conditions. This study focused on analyzing the strength of the drug from the perspective of the concentration- and pH-dependent self-aggregation of DOX in aqueous media. Dynamic light scattering (DLS) confirmed the strong effect of pH on the aggregation of drug particles. The concentration-dependent effect of DOX aggregation was depicted using circular dichroism (CD) and fluorescence spectroscopy. The change in the zeta potential of the aggregated forms of doxorubicin as a function of pH was determined. The Quartz Crystal Microbalance with Dissipation Monitoring (QCM-D) was used to form G4.0 PAMAM/DOX bilayers on the gold sensor, thus determining the most effective conditions for DOX binding to the G4.0 PAMAM structure. Depending on pH and drug concentration, the viscoelastic character of the formed layers was determined. Fluorescence spectroscopy determines the values of the binding constant. The Isothermal calorimetry (ITC) method indicated that DOX undergoes binding to the structure of G4.0 PAMAM in the endothermic process, localizing to two different types of active sites. In vitro tests for G4.0 PAMAM/DOX systems showed that the G4.0 PAMAM dendrimer can be a promising and selective nanocarrier for doxorubicin. The type of cancer cells strongly influences the efficiency of the formed complexes.

## 2. Results

### 2.1. Stability and Self-Aggregation of Doxorubicin under Alkaline Conditions

Since the PAMAM dendrimer efficiently binds doxorubicin under alkaline conditions (pH > 8.5) [29], the stability of the free drug was checked under this environment. UV-vis spectroscopy monitored the spectral changes in doxorubicin in relation to pH (pH = 8.5, 9.0, 10.0) at 72 h (Figure S1). It was shown that the tendency of changes in the position of spectra and absorbance values strongly depends on the pH of the initial solution. In the case of the solution at pH = 8.5, the trend of changes shows no significant effect on the doxorubicin form. The spectrum at pH = 8.5 shifts slightly towards higher wavelengths while it returns to the initial state after time. Absorbance increases with time and, after 2 h, is close to the native solution. The final pH of the doxorubicin solution remains at pH = 6.6 and is already constant after 1.5 h. A slower pH decrease with time was observed at pH = 9.0. The spectrum shifts slightly towards higher wavelengths, and the absorbance decreases relative to the native solution. A reduction in pH over time is observed, but at a slower rate than in the case of a solution with pH = 10. The trend of changes in the absorbance over time is different from that of the other solutions. For the first 2.5 h, its value increases, indicating the gradual return of the doxorubicin molecule to its initial form, while after this time, it begins to decrease gradually. Finally, after 24 h, the solution reaches a constant pH of pH = 7.07, while the absorbance is much lower than for the initial form at native pH. The most significant changes are observed for a solution at pH = 10. The spectrum shifts towards higher wavelengths at pH = 10 compared to initial conditions (pH = 5.44). The time effect makes the pH decrease reasonably fast, resulting in the range returning towards the initial spectrum while the absorbance decreases. After 24 h, no further changes were observed, and the pH of the solution gradually dropped to pH = 7.87. The DOX spectrum at pH = 10.0 is characterized not only by a significant shift and decrease in absorbance but also by the appearance of three characteristic spectral maxima due to the presence of different tautomeric conformers in solution. In this case, we observe the same peak position while the spectrum gradually returns to its initial position after 24 h. The disappearance of the three characteristic peaks is associated with the successful return of deprotonated forms to the initial state due to decreased pH. The molecule’s tendency to adopt different states in an aqueous solution at pH 7.5–10.0 causes the self-aggregation process of doxorubicin. Both the hydrophilic sugar part of daunosamine and the hydrophobic part of anthracycline promote aggregation of the DOX molecules in different environments, similar to other amphiphilic molecules [5,30]. DLS measurements confirmed the tendency for aggregation for DOX aqueous solution with a concentration of c = 0.5 mg/mL (Table S1).

**Figure 1:**
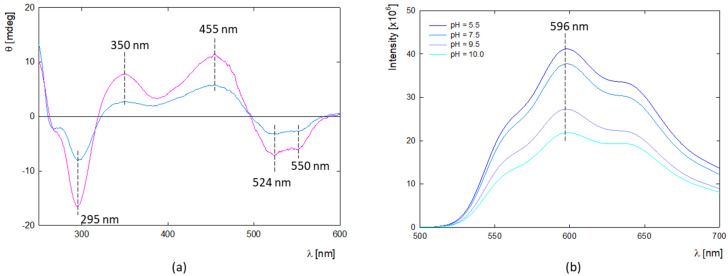
(**a**) CD spectra of aggregated forms of doxorubicin in water solution and its dependence on concentration (blue line—c = 0.5 mg/mL, pink line—c = 1.0 mg/mL). (**b**) Fluorescence spectra of doxorubicin and its dependence on pH (c = 2 μg/mL, H_2_O).

Circular dichroism (CD) was used to determine the structure of DOX molecules in aqueous solution at high concentrations (Figure 1a). CD spectra are characterized by the presence of two maxima at λ_max1_ = 350 nm and λ_max2_ = 455 nm and three minimum peaks at λ_min1_ = 295 nm, λ_min2_ = 542 nm, and λ_min3_ = 550 nm. The presence of two characteristic minima at λ_min2_ = 542 nm and λ_min3_ = 550 nm confirms DOX oligomerization in the given concentration range, which correlates well with the DLS results. This is supported by the results of Fülöp et al., which show that DOX aggregation occurs in aqueous solution above c = 0.5 mg/mL [30]. Fluorescence spectroscopy can be a valuable method for determining the potential DOX aggregation process. Doxorubicin exhibits fluorescence properties with a quantum yield of 4.5% in an aqueous solution [31]. It is worth mentioning that the drug in the monomeric form shows fluorescence properties with characteristic peaks with emission around λ_em_ = 560, 594, and 638 nm (φ_monomer_ = 3.9 × 10^−2^). At the same time dimerization occurs, the fluorescence yield decreases significantly (φ_dimmer_ ≈ 10^−5^) [32,33].

The fluorescence spectrum of DOX shows three distinct peaks at around λ_max1_ = 550, λ_max2_ = 596, and λ_max3_ = 640 nm (Figure 1b). The vibrational structures observed in the absorption and fluorescence spectra are due to symmetric modes associated with the dihydroxyanthraquinone molecule’s C=O bending, backbone stretching, and -OH bending motions [32]. The spectra’s shape and maximum position confirm the presence of the monomeric form of DOX at low concentrations. The trend of fluorescence intensity changes with pH shows that the protonated form of DOX exhibits the highest fluorescence, while above pH 7.5, it is successively quenched.

**Figure 2:**
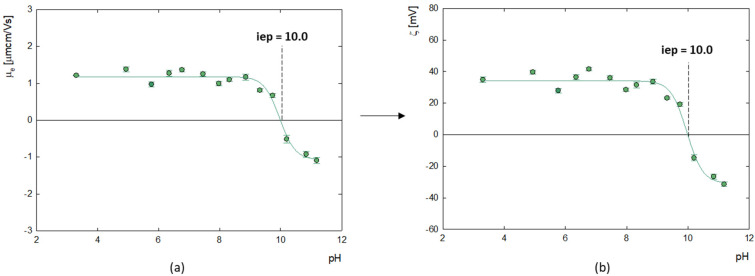
(**a**) Change in electrophoretic mobility (μ_e_) and (**b**) zeta potential (ζ) of aqueous solution of aggregated forms of doxorubicin as a function of pH with determined value of isoelectric point (iep) (c_DOX_ = 0.5 mg/mL).

The measurement of the electrophoretic mobility of doxorubicin molecules made it possible to determine their zeta potential, as illustrated in Figure 2. The analysis showed that the isoelectric point (iep) at which the molecules have a neutral charge (ζ = 0 mV) occurs at pH = 10.0. Below this value, the zeta potential of doxorubicin molecules is positive, which averages ζ = 35.8 ± 3.8 mV, while above this, at strongly alkaline pH, doxorubicin molecules become negatively charged. This correlates well with the results of Ryzkhina et al., who also observed a cationic form with a charge of ζ = 17.0 mV for an aqueous solution of DOX [34].

### 2.2. Physicochemical Properties of G4.0-DOX Complexes

#### 2.2.1. QCM-D and MP-SPR Studies

**Figure 3:**
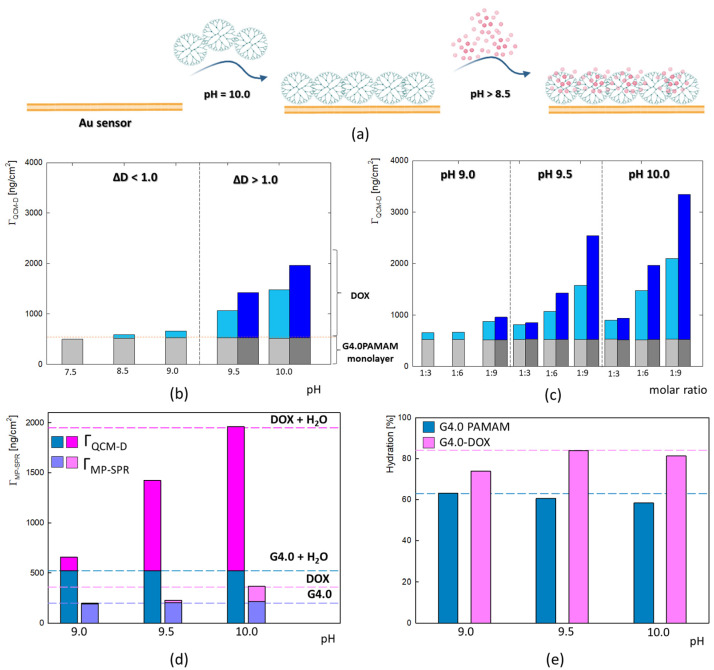
(**a**) Schematic representation of the G4.0-DOX bilayer formation on the Au surface (created with BioRender.com). (**b**) The mass of the adsorbed G4.0 PAMAM/DOX bilayers (*Γ_QCM-D_*) on the gold surface and its dependence on DOX pH, monitored by QCM-D (G4.0/DOX molar ratio 1:6, c_G4.0_ = 17.6 μM, pH_G4.0_ = 10.0, water). The gray color corresponds to the G4.0PAMAM monolayer, and the blue color corresponds to the DOX layer. The Sauerbrey model was used in the pH = 7.5–8.5 (light gray and light blue bars), and the Voigt model for pH = 9.0–10.0 (dark gray and dark blue bars). (**c**) The mass of the adsorbed G4.0 PAMAM/DOX bilayers on the gold surface and its dependence on DOX pH and concentration, monitored by QCM-D (c_G4.0_ = 17.6 μM, pH_G4.0_ = 10.0, water); with the exception of G4.0-DOX 1:3 pH = 9.0, the Sauerbrey and Voigt model was applied and compared to all the layers. (**d**) A comparison of the adsorbed masses by the QCM-D and MP-SPR methods. (**e**) The percentage of water in the QCM-D layers for the G4.0 PAMAM and DOX layers for the various pH conditions of the DOX solution (c_G4.0_ = 0.25 mg/mL, pH_G4.0_ = 10.0, c_DOX_ = 0.06 mg/mL, H_2_O).

The QCM-D method was used to determine the binding efficiency of doxorubicin to the G4.0 PAMAM dendrimer surface under physiological pH conditions (pH = 7.5) and alkaline conditions (pH = 8.5–10.0) (Figure 3b). For this purpose, a stable dendrimer monolayer was formed on the gold surface at pH 10.0 with the highest adsorption efficiency [35,36]. At each measurement, a steady and repetitive dendrimer monolayer with a thickness of d = 4.5 nm was formed, making it possible to compare the efficiency of DOX adsorption as a function of pH and concentration (Figure 3a). The layers where the G4.0 PAMAM/DOX molar ratio was 1:3 and 1:6 at pH 9.0 and 1:6 at pH 7.5 and 8.5 were characterized by dissipation < 1.0 × 10^−6^. Higher dissipation values and a lack of overtone overlap were observed for the other bilayers at alkaline pH and higher DOX concentrations (Figure S3). The G4.0 PAMAM/DOX bilayers exhibit a rigid structure for low concentrations and pH values. In contrast, the layers have viscoelastic properties for higher molar ratios and stronger alkaline pH. The properties of doxorubicin determine the nature of the formed bilayer. Under physiological pH, no adsorption of the drug on the G4.0 PAMAM surface is observed; there is a slight wash-off and loss of layer mass by about 20 ng/cm^2^. A strong effect of pH on the adsorption efficiency of doxorubicin on the G4.0 PAMAM surface is observed, which increases with increasing pH. The gradual increase in DOX adsorbed mass begins at a slightly alkaline pH (pH = 8.5). This is related to the change in the protonation of the DOX molecule. The tautomeric forms of doxorubicin that occur at alkaline pH have a negative charge, which can promote electrostatic binding to the dendrimer’s surface groups [37]. The most significant adsorption is observed when the dendrimer is electrostatically neutral (pH 10.0). Based on the literature data, the hydrolysis of DOX and a strong tendency to dimerization is observed in this condition [37]. The strong deprotonation of drug molecules may determine the binding efficiency of the protonated dendrimer groups. On the other hand, the deprotonation and subsequent formation of DOX dimers can lead to association with the dendrimer surface while forming aggregates, resulting in a strong increase in mass with increasing pH. Considering this effect, the influence of DOX concentration on adsorption efficiency under alkaline conditions (pH 9.0–10.0) was tested. Prepared drug concentrations corresponded to G4.0/DOX molar ratios of 1:3, 1:6, and 1:9. As shown by the results in Figure 3c, a strong effect was observed not only of increasing the pH of the drug on the adsorption efficiency but also of increasing its concentration. Thus, the highest layer was obtained for conditions when the pH of DOX was 10.0, and the molar ratio was 1:9.

The adsorption of analogous bilayers using MP-SPR made it possible to determine the degree of the hydration of the structure. The observed trend of mass increase as a function of pH is analogous for both methods. In the case of QCM-D, the sensor’s surface excess is much higher than MP-SPR (Figure 3d). This difference is due to the presence of water, which is detected in the QCM-D method. To determine the amount of water in the adsorbed layers, the degree of the hydration of the layers of three selected solutions was defined as a function of pH (pH = 9.0, 9.5 and 10.0, a molar ratio of G4.0/DOX = 1:6). The use of complementary QCM-D and MP-SPR methods made it possible to demonstrate the contribution of water to the mass of the formed layers. The G4.0 PAMAM monolayer has a hydration level of 58% at pH 10.0 to 63% at pH 9.0 (Figure 3e). The relatively low degree of the hydration of G4.0 PAMAM is due to the low level of the protonation of the internal and external groups of the dendrimer at high pH values. How it was presented for the G6.0 PAMAM hydration degree is strongly connected with pH (83% for pH = 9.0 and dropped to 64% at pH = 10.0)[38]. A successive increase in the amount of DOX adsorbed on the surface of the dendrimer leads to an increase in water content and results in a change in the hydration of the layer up to 82%. The number of DOX per carrier molecule (N_DOX_/1 G4.0 particle) resulting from the SPR mass of the layers was calculated. The results showed the incorporation of about 1 to 17 DOX particles per one G4.0 PAMAM particle, depending on pH. The highest efficiency complex formation was obtained for pH = 10.0 (Table 1). The results for both methods confirm the binding of DOX to the dendrimer structure under alkaline conditions. The successful functionalization of the gold sensor with a PAMAM dendrimer indicates its potential as a biosensor for quantifying bioactive molecules in biomedical applications.

#### 2.2.2. Fourier Transform Infrared (FTIR) Measurement

The formation of the stable complexes of dendrimer with doxorubicin is confirmed by FTIR spectra (Figure S5). The dendrimer spectrum (blue line) shows two main peaks at 1652 cm^−1^ and 1560 cm^−1^, which were assigned to an amide linkage. The peak at 3284 cm^−1^ was allocated to the primary amines present on the surface of the PAMAM structure [39,40]. The DOX spectrum (dark blue line) is characterized by the following peaks: 991 cm^−1^ and 1023 cm^−1^, which are given to the skeletal ring. The following four peaks at 1083 cm^−1^, 1117 cm^−1^, 1214 cm^−1^, and 1288 cm^−1^ correspond to C-N, C-O-C, and C-O stretching vibrations. The subsequent peaks at 1414 cm^−1^ and 1443 cm^−1^ were assigned to methyl and hydroxyl group bending vibration, respectively. The peak at 1580 presents N-H bending and C-N stretching vibrations^41^. The FTIR spectra of the G4.0-DOX complexes confirm the interactions between the components. Despite maintaining a constant dendrimer concentration in the complexes, the signals at 1562 cm^−1^ and 3289 cm^−1^ are enhanced. A shift of the signal at 1562 cm^−1^ to 1577 cm^−1^, originating from internal amide groups, is observed. The peak from the surface amine groups at 3289 cm^−1^ thickens and shifts to 3284 cm^−1^, confirming interactions with the drug molecule. In addition, the complexes show a drug-specific peak at about 3500 cm^−1^, attributed to water molecules bound to drug molecules [41].

#### 2.2.3. Binding Constants and Thermodynamic Parameters of G4.0-DOX

**Figure 4:**
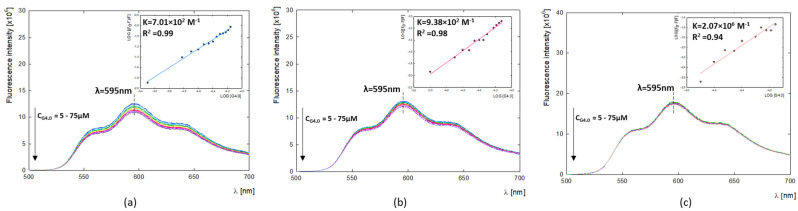
Fluorescence emission spectra of G4.0 PAMAM-DOX systems presented for: (**a**) G4.0-DOX at pH 7.5; (**b**) G4.0-DOX at pH 9.0; (**c**) G4.0-DOX at pH 9.5 and LOG((F0-F)/F) versus LOG[G4.0] plots.

The fluorescence properties of DOX can help study carrier–drug interactions and imaging in biological systems [42,43]. In the present study, the fluorescent properties of doxorubicin were used to determine G4.0-DOX binding constants (*K_a_*) by fluorescence quenching through the presence of a dendrimer (Figure 4). The values of the binding constants equal K_7.5_ =2.07 × 10^6^, K_9.0_ =9.38 × 10^2^, and K_9.5_ = 7.01 × 10^2^, and their values decrease with increasing pH. The determined values of the binding constants at alkaline conditions are K_9.0_ = 9.38 × 10^2^ M^−1^ and K_9.5_ = 7.01 × 10^2^ M^−1^, and they are close to the values that we obtained in previous work using UV-vis with Hill’s method (K_9.0_ = 2.39 × 10^2^ and K_9.5_ = 2.73 × 10^2^) [29]. The value of the binding constant at pH 7.5 is close to that obtained by Chanphai et al. for the same system in a PBS buffer at physiological pH as well (K = 1.6 × 10^6^) [44]. In contrast, the G4.0-DOX system at physiological pH was the only one among the studied systems for which the thermodynamic parameters could be determined using calorimetric studies. The ITC measurements were performed for the G4.0-DOX system in the PBS buffer at pH = 7.4. Measures at alkaline pH in water were impossible due to the high concentration of DOX in the syringe and the associated strong aggregation. Calorimetric measurements indicate that the G4.0 PAMAM binds DOX in the endothermic process (ΔH > 0) with a significant positive entropy change. All the thermodynamic parameters obtained for the G4.0PAMAM/DOX system are collected in Table 2. An independent model is used when the multiple binding sites on a macromolecule are present and are independent. Referring to the shape of the obtained isotherm, it can be assumed that the binding sites are not identical (Figure S6). The best fit for the results obtained is a multiple-site model that may be related to non-identical binding sites or interactions [45]. Binding sites have different affinity; while all the binding sites are similar, it could be a cooperative process. The first binding site (K_a1_ = 1.215 × 10^5^ M^−1^; K_d1_ = 8.232 × 10^−6^ M) had a stronger association than the second one (K_a2_ = 1.065 × 10^2^ M^−1^; K_d2_ = K_d1_ =9.390 × 10^−3^ M). The enthalpy of site 1 is ΔH_1_ = 9.068 kJ/mol, and the poorer binding at site 2 was entirely attributed to a less favorable enthalpy (ΔH_2_= 198.9 kJ/mol). Considering all the parameters, the change in enthalpy, the difference in entropy, and the change in Gibbs free energy, it can be seen that in the system under study, the reaction occurs spontaneously with the preferred contribution of the entropic factor. The enthalpy change is much lower than the −TΔS value, which indicates a spontaneous process of binding DOX to G4.0 PAMAM at the studied temperature [46]. The binding enthalpies for the considered two binding sites are positive, and their values equal ΔH_1_ = 9.068 and ΔH_2_ = 198.9 kJ/mol, respectively. The binding entropies are also positive with values ΔS_1_ = 1.278 × 10^2^ J/mol·K and ΔS_2_ = 7.058 × 10^2^ J/mol·K. Rise entropy value indicates that the system becomes more disordered. The most preferred effect of the G4.0-DOX interaction is the effect of desolvation (hydrophobic force) and conformational changes [47]. This is most likely due to the release of dissolution molecules surrounding the DOX molecules and filling the binding sites of the G4.0 PAMAM dendrimer.

#### 2.2.4. Electrophoretic Mobility

**Figure 5:**
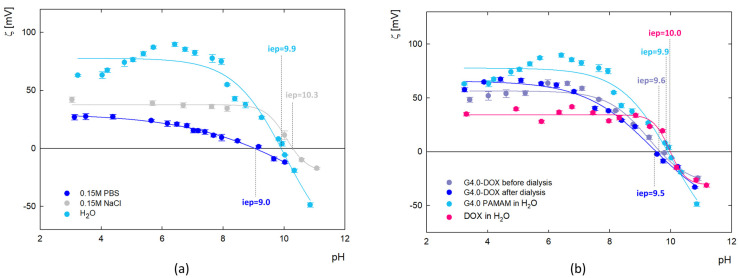
Change in the zeta potential (ζ) of the G4.0 PAMAM dendrimer depending on the ionic strength and solvent (**a**) and complexes with doxorubicin before and after dialysis (**b**) with the determined values of isoelectric point (iep) (H_2_O, molar ratio 1:6, pH 9.5).

The values of zeta potential as a function of pH for the dendrimer and the G4.0 PAMAM-DOX complex were calculated from electrophoretic mobility (μ_e_) measurements and presented in Figure 5. The results show changes in the zeta potential of G4.0 PAMAM as a function of pH, ionic strength, and solvent (Figure 5a). The presence of amine functional groups on the surface of the dendrimer determines the charge changes. In the aqueous solution, G4.0 PAMAM has a high zeta potential (69.7 mV for pH = 7.5), which successively decreases with increasing pH and reaches the isoelectric point (iep) at pH = 9.9. Increasing the ionic strength causes the zeta potential to drop to 37.7 mV for 0.15 M NaCl and 13.9 mV for 0.15M PBS (pH = 7.5), consistent with the other literature data [48]. Depending on the solvent, the isoelectric point is shifted to pH_iep_ = 10.3 and pH_iep_ = 9.0 for NaCl and PBS, respectively.

The zeta potential of the complex (Figure 5b) was measured immediately after the complex formation and after the dialysis process. Dialysis was used to remove DOX molecules that were not bound to the carrier. Slight changes in the zeta potential value generally characterize the course of the curves before and after dialysis. In the pH range of 4–6, an increase in the value of the zeta potential after dialysis is observed concerning the initial zeta potential of the complex. In this pH range, DOX exists in a protonated form, thus being released from the complex due to electrostatic repulsion. These measurements confirm the immobilization of the DOX molecules on the surface of the dendrimer structure in a reversible manner.

### 2.3. Cytotoxicity and Release Studies

#### 2.3.1. In vitro MTT Assay

**Figure 6:**
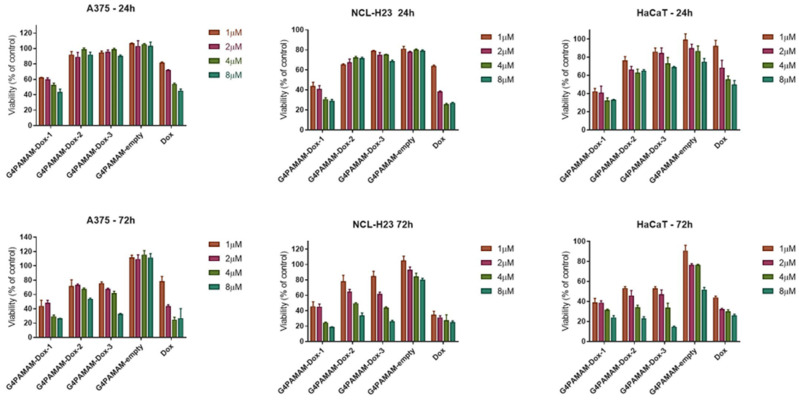
The cytotoxic effect of G4.0 PAMAM nanocarriers against human cancer (A375, NCL-H23) and immortalized cells (HaCaT) as a model of the normal cell line, evaluated by the MTT assay after 24 and 72 h.

Doxorubicin is a commonly used chemotherapeutic agent, and it was used as a model drug for validating nanosystems to deliver drugs depending on the type of cell [13]. The nanosystems based on dendrimers improved targeting, controlled release, enhanced bioavailability, and reduced organ toxicity, making them promising candidates for cancer treatment [49]. Zhang et al. also conjugated PAMAM dendrimers with doxorubicin, modified these nanocarriers by folic acid (FA), and showed anticancer effects against epidermoid carcinoma (KB cells) [50]. Marcinkowska et al. used PAMAM dendrimers conjugated with doxorubicin and monoclonal antibody (trastuzumab) versus breast cancer cells. The authors demonstrated that PAMAM-dox-trastuzumab conjugate was more effective for tumors with HER-2 overexpression—here SKBR-3 cells [51]. In the present study, in vitro tests for dendrimers and their complexes with doxorubicin were conducted against immortalized keratinocytes (HaCaT) as a model of the normal cell line and six cancer cell lines (A375, HT-29, NCL-H23, LoVoDX, MCF-7, and MCF-7/DX). According to our previous experience, we have selected 24 and 72 h, commonly chosen endpoints for cytotoxicity assays that allow researchers to capture variations in cellular response over time. More extended incubation periods, such as 72 h, provide insights into whether cytotoxic effects persist, diminish, or escalate over time. This information is crucial for evaluating substances’ safety profile, especially those intended for prolonged exposure, such as pharmaceuticals or environmental pollutants. Cytotoxicity tests showed no cytotoxicity of G4.0 PAMAM-empty dendrimers against normal and cancer cells (Figure 6). The selective anticancer activity of the G4.0 PAMAM-DOX-1 complex was observed against A375 human skin cancer cells and NCL-H23 human lung cancer cells, as demonstrated in Figure 6. No significant cytotoxic effect was observed in the case of drug-resistant cells MCF-7/DX and LoVoDX (Figure S7). In the case of both breast cancer cell lines (sensitive and resistant), G4.0 PAMAM-DOX-3 for 8 µM induced a significant viability decrease below 40%. In turn, keratinocytes were more sensitive to G4.0 PAMAM-DOX-1 after 24 h exposure, and after 72 h, all the nanocarriers induced a similar cytotoxic efficacy. The results confirmed the effectiveness of the dendrimers and G4.0 PAMAM-DOX complexes firmly on cell type. In some cases, the therapeutic effect of DOX can be enhanced by the presence of G4.0 PAMAM dendrimers. To extend the analysis of anticancer activity, the IC50 dose for free DOX and G4.0-DOX complexes was determined (Table 3). The calculation of the IC50 compound was possible only for the cell lines for which significant cytotoxicity was observed, i.e., normal keratinocytes (HaCaT), malignant melanoma (A375), and lung cancer (NCl-H23). After the 24 h incubation of keratinocytes with free DOX, the IC50 value was 1.57 μg/mL (2.61 μM). A similar value after 24 h of incubation with the same line was recorded by Pessila et al. (IC50 = 2.72 μM) [52]. After 72 h of incubation, doxorubicin’s IC_5_0 drastically decreased about four times and was 0.37 ug/mL (0.58 μM). To determine if any of the tested compounds are more selective towards cancer cell lines than normal keratinocytes, the selectivity index (SI) was calculated based on the obtained IC_50_ values. SI is a pure IC_50_ value ratio in a non-cancer cell line (HaCaT) to IC50 value in a cancer cell line (A375 and NCl-H23). Study demonstrates the poor selectivity of free DOX towards malignant melanoma (SI = 0.29) and lung cancer cells (SI = 0.61). An improvement in selectivity was noted when DOX was complexed with G4.0 PAMAM at a molar ratio of 1:12 and pH = 9.5, where its selectivity was increased by 147 % (SI = 0.79) and 21% (SI = 0.81) against malignant melanoma and lung cancer, respectively.

#### 2.3.2. G4.0 PAMAM Internalization In Vitro

**Figure 7:**
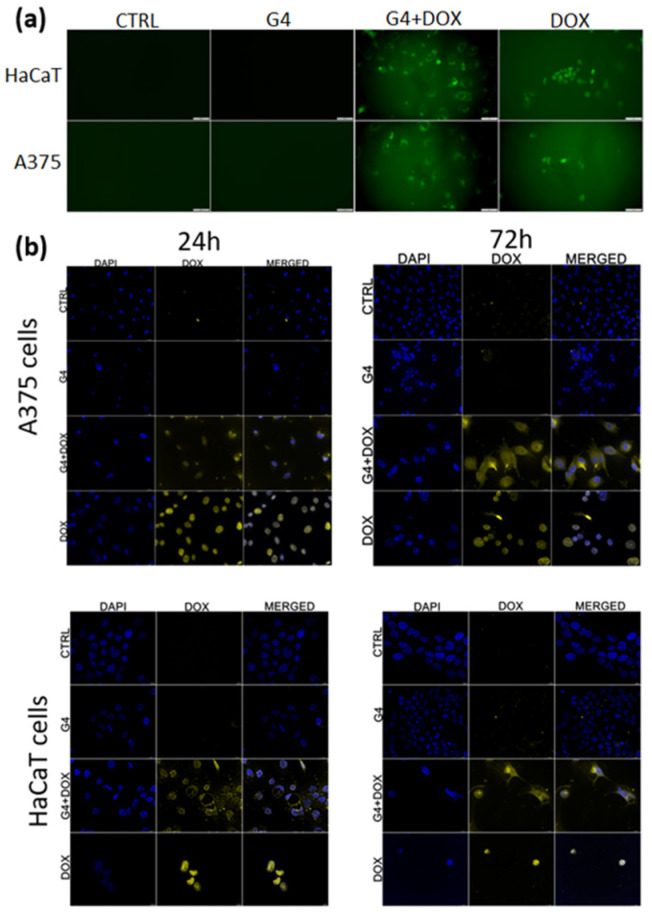
Internalization of G4.0 PAMAM-DOX and free DOX by confocal microscopy in A375 and HaCaT cells: intravital analysis after 24 h (**a**) and analysis of fixed cells after 24 and 72 h (**b**). DOX- green color; nuclei- DAPI blue color.

The imaging presented in Figure 7 represents the intracellular distribution of the free or encapsulated doxorubicin in the A375 and HaCaT cells. Figure 7a shows the distributions of the nanosystems in living cells post 24h after exposure with a clear difference between the free DOX and DOX loaded in the nanosystems. The free doxorubicin is localized in nuclei and incorporated in the G4.0 PAMAM systems, and it is distributed in the whole cytoplasm of both treated cell lines. The colocalization imaging of the fixed cells with the nanosystems and stained nuclei confirmed the distribution of doxorubicin in the cancer cells and partially in the normal cells. In turn, G4.0 PAMAM-DOX is distributed in the cytoplasm and nuclei. However, in the case of the normal cells, after 24 h, we could also observe nuclear DOX distribution, and the post 72 h DOX in the nanosystems was translocated to the cytoplasm (Figure 7b).

**Figure 8:**
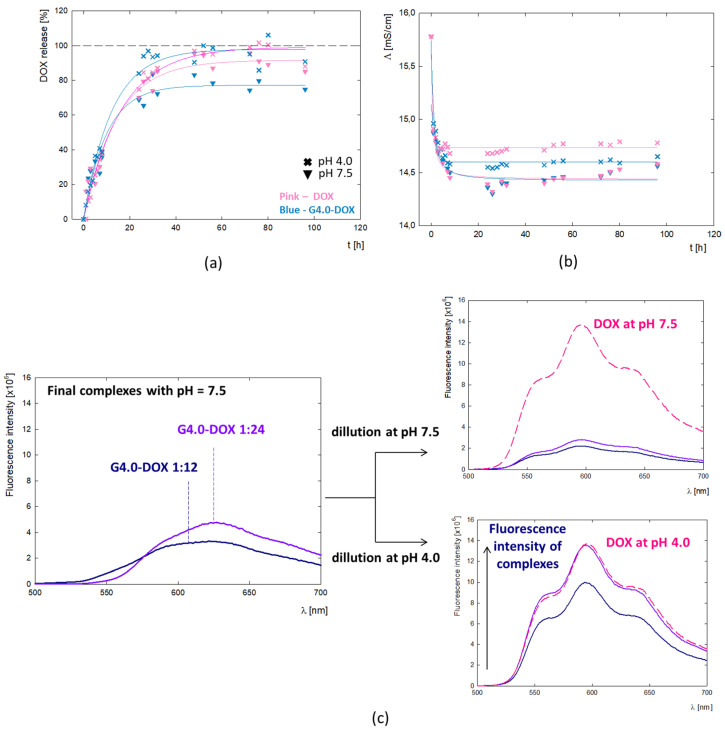
(**a**) Release profile of doxorubicin from the complex compared to the DOX of the same concentration (c = 38 μM) into the PBS buffer at pH 7.4. Triangle—final complex at pH 7.5; cross—final complex after reducing to pH = 4.0. (**b**) Change in the electrolytic conductivity of the PBS buffer during the release assay. (**c**) The fluorescence spectra of the G4.0-DOX complexes at the molar ratios of 1:12 and 1:24, their changes after dilution in water at pH 7.5 and pH 4.0 and comparison with the reference DOX solution at the same pH and concentration (c = 5 μM).

To better understand the behavior mechanism of the G4.0-DOX at the cellular level, the profile of DOX release from the complex was determined (Figure 8a). The tests were conducted in two variants—for the final G4.0/DOX complex at pH 7.5 and after reducing its pH to 4.0. In the presented DOX release profile, two main process steps are observed: a rapid release of the active substance during the first 24 h of incubation and a slow process for the next 72 h. This may be related to the location of the molecules in the dendrimer structure and thus to a change in interactions and, consequently, the method of drug release from the carrier. It is expected that the molecules located on the surface of the carrier can be released into the environment without a barrier. In the case of the molecules contained inside the structure, their release may be difficult due to the steric interactions resulting from the level of the protonation of the functional groups present in the carrier itself. This confirms the gradual release of DOX from the complex. After 24 h, 60% of the drug is released, while 70% is released after 72 h. The two-step mechanism of drug release from the polymer structure has a positive therapeutic effect as it prolongs the drug release. The drug bound to the dendrimer remains in the cytoplasm, where it also plays its cytotoxic role. The DOX release rate up to 96 h is identical regardless of pH.

Based on the doxorubicin release curves, the kinetic parameters of the process were determined (Table 4). The value of the rate constant for the release of DOX from the complex (k_1_) is similar regardless of pH. The amount of DOX released per hour equals 8.2% and 9.1% at pH = 4.0 and pH = 7.5, respectively. A significant difference is observed at the final stage of the process, where the maximum percentage of the released drug A_max_ = 73.3% and A_max_ = 97.9% of the active substance is released from the complex at pH = 7.5 and pH = 4.0, respectively. This apparent difference may be due to the overall dissociation of DOX from the complex due to the change in ionization form at pH = 4.0. The fluorescence spectroscopy revealed the fluorescence quenching of the drug upon binding to G4.0 PAMAM. At pH = 7.5, despite the strong fluorescence of the reference system being the DOX solution, no signal from the drug is observed in the spectrum of the complexes. When the pH of the G4.0PAMAM-DOX system is lowered to pH = 4.0, a peak appears in the spectrum with emission at λ_em_ = 596nm, characteristic of the fluorescence emission from doxorubicin (Figure 8c). At pH 4.0, the doxorubicin enclosed inside the complex is released. The mechanism of drug release from the carrier structure in an acidic environment is also confirmed by release tests.

## 3. Discussion

Doxorubicin can take different tautomeric forms—protonated and deprotonated depending on the pH of the solution. Under physiological conditions, the predominant form is protonated with a positive charge on the amino group [53]. Its presence is directly related to the mechanism of action and DOX-DNA interaction, which is stabilized, i.e., by electrostatic interactions between the cationic amino sugar of doxorubicin and the anionic phosphate backbone of DNA [54]. The stability studies of doxorubicin as a function of pH and time conducted using UV-vis spectroscopy showed that depending on the conditions, a part of the drug concentration is degraded. The spectrum of the drug at pH = 10.0 differs significantly from the others, where three characteristic maxima are observed. Florencio et al., using theoretical methods, investigated the effect of four DOX tautomers and their deprotonated forms as a function of pH on doxorubicin’s spectral profile. They showed that the spectrum of DOX under alkaline conditions (pH = 10.08) is characterized by three peaks around 500, 550, and 600 nm, corresponding to the simultaneous contribution of the four deprotonated conformers [55]. Doxorubicin’s tendency to self-aggregation under physiological conditions may be responsible for its cytotoxicity, cellular immunity, or reduced anticancer activity. The tendency for the drug to self-aggregate was confirmed for concentration > 0.5 mg/mL, and the process was shown to intensify at pH > 9.0, which is related to the presence of various DOX conformers with opposite charges in the aqueous solution. Fülöp et al. pointed out the effect of DOX concentration on self-aggregation in an aqueous solution using the permeation technique and showed that dimers and trimmers were observed at deficient concentrations of doxorubicin. At a concentration of 0.5 mg/mL (8.6 × 10^−3^ M), aggregates containing about 40 molecules were present [30]. In contrast, using circular dichroism (CD) spectra, Anand et al. demonstrated the presence of DOX dimers in phosphate buffer at pH 7.4 at a concentration ≥ 1.0 × 10^−5^ M [56], while Maruf et al. report a limit of 6.9 × 10^−5^ M, above which DOX begins to aggregate in PBS buffer at pH 7.4 [3]. In the present study, the effect of pH changes on the spectrum of doxorubicin at low concentrations (2 μg/mL) was performed using fluorescence spectroscopy. The spectra’s shape and maximum position (λ_em_ = 596 nm) confirm the presence of the monomeric form of DOX at low concentrations [33]. As has already been demonstrated in the literature, DOX dimerization or polymerization can strongly disrupt its fluorescence, leading to a significant reduction in intensity and a bathochromic shift in the spectrum [32,33]. In our case, a change in the spectrum is not observed, proving the absence of DOX polymers at the studied concentration and pH. The significant reduction in intensity with increasing pH may be due to the partial degradation of the drug and the subsequent extinction of its fluorescence.

The critical issue that was studied in the present work is the effect of the drug molecule’s charge and form on interactions with the dendrimer carrier. Our previous work demonstrated that G4.0 PAMAM can efficiently bind DOX in an alkaline environment (pH > 9.0) with a loading capacity of LC = 4.80–40.16 % depending on conditions [29]. Liping et al. determined the efficiency for the same system in the aqueous environment at LC = 8.1–8.8% [17]. Also, many other works are focused on developing an effective DOX delivery system based on dendrimer nanocarriers [57,58]. Changes in the electrophoretic mobility values of the formed complex and FTIR spectroscopy monitored the successive immobilization of DOX to the G4.0 PAMAM structure. Using the QCM-D method, the influence of pH on the efficiency of the formation of the G4.0 PAMAM-DOX complex in dynamic conditions was tested. The effective adsorption of DOX to the dendrimer layer was observed for pH > 8.5, and the highest for pH = 10.0. Both pH and drug concentration have a significant impact on the viscoelastic properties of the formed G4.0/DOX bilayer. At pH < 9.0 and in the range of low drug concentrations (molar ratio G4.0/DOX 1:3), the bilayers formed are stiff. Bilayers formed at higher DOX concentrations and pH ≥ 9.0 show an increase in viscoelastic properties. Based on the QCM-D and MP-SPR measurements, the degree of the hydration of both the G4.0 PAMAM monolayer (58–63% at pH 9.0–10.0) and the G4.0/DOX bilayers (72–82% at pH 9.0-10.0) was determined. The presence of drug molecules causes a significant increase in the system’s hydration. The affinity of the drug to the dendrimer was determined under basic conditions using fluorescence spectroscopy (K_7.5_ = 2.07 × 10^6^, K_9.0_ = 9.38 × 10^2^, K_9.5_ = 7.01 × 10^2^). The course of the ITC isotherm indicates that two different types of active sites, differing in affinity, are involved in forming the complex with doxorubicin (K_a1_ = 1.215 × 10^5^ M^−1^, K_a2_ = 1.065 × 10^2^ M^−1^). The ΔG value indicates that we are dealing with an endothermic process. Since the ΔS and ΔH parameters have positive values, the hydrophobic interaction has a significant contribution to the formation of the complex. Under basic conditions, the dominant form of doxorubicin is the tautomeric form with a negative charge, which, through electrostatic interaction, is immobilized on the surface of the carrier, which under these conditions has a positive charge. It should be noted that other tautomeric forms also occur under these conditions and may prefer to be located in the hydrophobic interior of the polymer.

In vitro studies have shown a specific anticancer activity of the G4.0-DOX complexes and increased selectivity towards malignant melanoma and lung cancer cell lines. Doxorubicin contained in the complex is less toxic than free DOX towards the HaCaT cell line. A significant decrease in HaCaT cell viability is due to the fact that they exhibit high levels of topoisomerase I activity, which is significantly inhibited by doxorubicin [52]. The complexation of doxorubicin with a dendrimer carrier decreased its IC_50_ against HaCaT cells by up to more than two times, indicating protection from the drug’s harmful effects on normal cells. In addition, a destructive impact of free DOX on the HaCaT cell membrane is observed, while binding to the carrier significantly reduces this effect, proving less harmful interference of the complex against keratinocytes. The dendrimer-bound drug localizes to the nucleus and the cytoplasm, while the free drug accumulates preferentially in the cell nucleus. The diversified distribution within the cell indicates a specific interaction of both forms at the molecular level, with no disruption of the therapeutic effect of the drug administered in the form of a complex. It should be emphasized that due to the structure of the dendrimer, doxorubicin molecules are released from the complex in two ways. The particles that are electrostatically immobilized on the surface of the carrier structure are easily released, while the molecules that are bound inside by hydrophobic interactions are more difficult to release. Based on the release curves, it was determined that at pH = 7.4, 73% of the drug was released, while at pH = 4.0, up to 100%. The release of a more significant amount of doxorubicin at acidic pH comes directly from the change in the protonation of the carrier, the opening of the polymer structures, and thus the availability of the drug molecules located in hydrophobic pockets. These findings support the pH-regulated mechanism of doxorubicin release from the G4.0-DOX complex. The other available studies also indicate that minimum micromolar DOX concentrations were active in cancer cells, e.g., loaded in polypeptide nanocarriers in lymphoma [59], when combined with L-Canavaine against HeLa, Caco-2, MIA PaCa-2, BxPC-3, HEP G2, and SK-HEP-1 [60], DOX in G4PEG against MCF-7 cells revealed significantly higher concentration 1.3 mM [61]. The PAMAM dendrimers in our study are highly branched, nanoscale polymers that can form complexes with DOX at various binding ratios. These ratios represent different levels of drug loading, which can influence the overall physicochemical properties of the complexes, including their size, charge, and drug release profile. Several factors could explain the observed trend of increasing IC_50_ values with higher DOX loading ratios: (1) drug release dynamics: At higher binding ratios (e.g., 1:6 and 1:12), the interaction between DOX and the dendrimer may result in a more stable complex, which could slow down the release of DOX. This slower release rate may lead to reduced immediate cytotoxicity, as reflected in higher IC50 values; (2) dendrimer saturation: As the binding ratio increases, the dendrimer surface may become saturated with DOX molecules. This saturation can lead to changes in the complex’s overall conformation and reduce the availability of free DOX to interact with cellular targets; (3) steric hindrance and accessibility: Higher DOX loading may cause steric hindrance, affecting the ability of the DOX molecules to access and penetrate the cancer cells effectively. This reduced accessibility can result in lower cytotoxicity; (4) aggregation and stability: At higher binding ratios, there is a potential for forming larger aggregates, which could alter the cellular uptake and distribution of the complexes. These aggregates might be less efficient in delivering DOX to the intracellular targets, leading to decreased cytotoxic effects.

## 4. Materials and Methods

### 4.1. Materials

Poly(amidoamine) dendrimers generation 4.0 (G4.0 PAMAM) was acquired from Dendritech, Inc. (Michigan; Midland, MI, USA), doxorubicin hydrochloride (DOX) from Ambeed (Arlington, IL, USA), and deuterium oxide (D_2_O, 99.9 atom % D) was supplied by Deutero GmbH (Kastellaun, Germany). The solutions were prepared in deionized water, and the pH was adjusted using sodium hydroxide (NaOH) and hydrochloric acid (HCl) from Chempur (Poland). The G4.0 PAMAM solutions for electrophoretic mobility measurements were additionally prepared in Dulbecco′s phosphate-buffered saline (PBS) and sodium chloride (NaCl) from Sigma-Aldrich (Saint Louis, MO, USA).

### 4.2. Dynamic Light Scattering (DLS)

The diffusion coefficient of the DOX in water solution was determined using a Malvern Nano ZS analyzer (Malvern, Worcestershire, UK). The self-diffusion coefficient (D) allowed us to calculate the hydrodynamic radius (R_H_) of the dendrimer using the Stokes–Einstein equation:

(1) $$R_{H}=\frac{k_{B}T}{6\pi \eta D}$$

where *k_B_* is the Boltzmann constant, *T* is the absolute temperature, and *η* is the viscosity of the solution. The doxorubicin concentration was 0.5 mg/mL. The pH of the solution was adjusted using sodium hydroxide (NaOH) with a concentration of c = 0.1 M.

### 4.3. Quartz Crystal Microbalance with Dissipation Monitoring (QCM-D)

The adsorption process of the G4.0 PAMAM/DOX bilayers on the gold (Au) surface was carried out using the Q-Sense E1 device (Biolin Scientific, Finland). During the measurements, two parameters were monitored: the change in the resonance frequency (∆*ƒ*) and the change in energy dissipation (∆*D*). The first layer was formed by the G4.0 PAMAM dendrimer itself, whose aqueous solution was adsorbed each time under constant conditions (c = 17.6 µM, pH = 10.0). The DOX bilayer was adsorbed in two variants. The first variant was pH-dependent, while the second variant was concentration-dependent. The pH range was 7.5–10.0, while the drug concentration corresponded to the G4.0 PAMAM/DOX molar ratios of 1:3–1:9. For the rigid layers, the mass adsorbed on the sensor surface (*Γ_QCM-D_*) was calculated based on the Sauerbrey model for overtone n = 7 using the Qtools software. The mass adsorbed on the sensor surface for viscoelastic layers was calculated based on the Voigt model using the Qsense Dfind (Biolin Scientific, Espoo, Finland) software based on the measurement results for 3–11 frequency overtones.

The QCM-D technique assumes that when a substrate layer is adsorbed onto the sensor surface, the resonant frequency (*ƒ*) decreases with increasing adsorbed mass (*Γ_QCM-D_*) [62]. Rigid or viscoelastic properties characterize adsorbed layers and can be described by the Sauerbrey or Voigt models, respectively. When the adsorbed mass is uniformly distributed over the sensor surface and exhibits low energy dissipation (0–1.0 × 10^−6^), we can consider such a layer rigid [63]. Then, the frequency shift (Δ*ƒ*) is proportional to the adsorbed mass per unit area (Δ*Γ_QCM-D_*) in the Sauerbrey model [62,64]:

(2) $$\Gamma_{QCM-D}=C_{f}\frac{\Delta f}{n}$$

where *∆f*—changes in the resonance frequency (Hz), *C_f_*—constant characteristic for quartz crystals (*C_f_* = 17.7 ng/cm^2^), and *n*—number of overtones (in the present case *n* = 7).

The Kevin–Voigt viscoelastic models relate the measured dissipation factor (*D*), whose values exceed 1.0 × 10^−6^ [63,64]. In the Voigt model, multiple Δƒ and Δ*D* data obtained at several overtones are used to calculate the viscoelastic properties of the adsorbed layer [62]:

(3) $$G^{*}=G^{’\;}+iG^{\prime \prime }=\mu_{1}+i2\pi ƒ\eta_{1}$$

where *ƒ* is the resonant frequency, G* is the complex shear modulus, *G’* is the storage modulus, and *G’’* is the loss modulus [62]. The mass calculation using the Voigt model was performed using the Qsense Dfind software.

### 4.4. Multi-Parametric Surface Plasmon Resonance (MP-SPR)

The G4.0 PAMAM/DOX bilayer adsorption measurements on the gold surface (Au) were performed using the MP-SPR Navi^TM^ 200 apparatus (BioNavis Ltd., Finland), a goniometer coupled to a prism (Krechmer mode). The system has two separate channels, each of which can emit wavelengths of λ = 670 and λ = 785 nm. The MP-SPR apparatus worked in a wide angular scan range from 40 to 78°. All the experiments were performed at a constant flow rate of 50 μL/min. The immobilization of the molecules on the Au sensor surface is monitored by registering intensity changes in the set angle or changes in the resonance angle’s value over time. The excess of the mass of the adsorbed particles determined using MP-SPR (*Г_MP-SPR_*) on the gold surface is calculated utilizing the following equation [38,63]:

(4) $$\Gamma_{MP-SPR}=\frac{\Delta \Theta_{SPR}k}{\frac{dn}{dc}}$$

where $\Delta \Theta_{SPR}$ is the change in the MP-SPR angle, k is an MP-SPR instrumental constant, and dn/dc is the refractive increment; k=1 × 10^−7^ nm/deg for λ = 670 nm and dn/dc ≈ 0.239 cm^3^/g for the G4.0 PAMAM dendrimer.

The hydration degree of the layers was calculated from the following equation [38]:

(5) $$\Gamma^{H_{2}O}=\frac{\Gamma_{QCM-D}-\Gamma_{MP-SPR}}{\Gamma_{QCM-D}}\times 100\;\left[\%\right]$$

where $\Gamma_{MP-SPR}$ is the adsorbed mass from MP-SPR, $\Gamma_{QCM-D}$ is the adsorbed mass from QCM-D, and $\Gamma^{H_{2}O}$ is a fraction of water in the adsorbed film.

### 4.5. Fourier-Transform Infrared Spectroscopy (FTIR)

The Fourier-transform infrared spectroscopy (FTIR) measurements were performed using a Nicolet iS50, Thermo Fisher Scientific, MA/USA FTIR spectrometer with the SMART SAGA specular reflection (SR) accessory. The measurements were conducted on the samples adsorbed on the gold surface layer with a thickness of 100 nm deposited on the glass plate by vapor deposition. Measurements were made for the aqueous solutions of doxorubicin with the concentration of 0.24 mg/mL and the G4.0-DOX complexes prepared at molar ratios of 1:6, 1:12, and 1:24 and constant dendrimer concentration (c = 0.25 mg/mL). The encapsulation efficiencies for the tested nanosystems were, respectively: EE = 32.76%, EE = 35.82%, and EE = 40.16%. The pH of all the solutions was adjusted to pH = 9.5. For each sample, a drop of the solution was applied to a clean gold surface and allowed to evaporate under cover. The FTIR spectra were recorded in the wavenumber range of λ = 700 to 4000 cm^−1^. A total of 512 scans were averaged for each spectrum with a spectral resolution of 4 cm^−1^. Before each measurement, the background spectrum of the clean surface was recorded, and then it was automatically subtracted from the sample spectrum. The Omnic software (Thermo Fisher Scientific, MA/USA) was used for data analysis.

### 4.6. Electrophoretic Mobility Measurement

Electrophoretic mobility (µ_e_) was determined using a Malvern Nano ZS analyzer (Worcestershire, UK). All the electrophoretic mobility measurements were conducted in the pH range of 2.0–11.0. The zeta potential was related to the electrophoretic mobility via Henry’s equation. For the calculations, the Smoluchowski limit (*f(κa)* = 1.5) was applied for most of the solutions. Only for G4.0 PAMAM in water, the Hückel limit (*f(κa)* = 1.0) was used. Pure DOX solution was measured in water with a drug concentration of c = 0.5 mg/mL (c = 0.86 mM). Pure G4.0 PAMAM solutions were measured depending on the solvent: NaCl (I = 0.15 M) and PBS buffer (I = 0.15M, pH = 7.5) in deionized water. The dendrimer concentration was c = 1 mg/mL (70 μM). The G4.0 PAMAM–DOX complex measurements were made in water with a constant dendrimer concentration of c = 1 mg/mL (c = 70 µM). The complexes were prepared at G4.0/DOX molar ratio 1:6 and initial pH = 9.5 (EE = 32.76%,). The first measurement variant was for the complex without dialysis, while the second was for the complex after dialysis, where the drug particles not bound to the dendrimer were removed. The complexes were dialyzed using Slide-A-Lyzer™ Dialysis Cassettes (MWCO 10.0 kDa), Thermo Fisher (Waltham, MA, USA). The dialysis was conducted in deionized water for 24h in darkness. The pH of the solutions was adjusted using sodium hydroxide (NaOH) with a concentration of c = 0.1 M and hydrochloric acid (HCl) with a concentration of c = 0.05 M.

### 4.7. Circular Dichroism

The circular dichroism (CD) measurements were performed at a spectrometer (Jasco, MD, USA) with a 10nm cuvette. The CD spectra for the DOX solutions were recorded in water for the drug concentrations of 0.5 mg/mL (0.86 mM) and 1.0 mg/mL (1.72 mM). The spectra were recorded for two wavelength ranges of λ = 240–450 nm and λ = 250–650 nm with a resolution of 1 nm, while the scanning speed was equal to 50 nm/min.

### 4.8. Fluorescence Spectroscopy

The corrected fluorescence emission spectra of free Doxorubicin and G4.0-DOX complexes were obtained on a Fluorolog spectrofluorometer (HORIBA Jobin Yvon Ltd., Middlesex, UK) using 1 nm resolution, with both the emission and excitation monochromator slits set to 5 nm. Samples in plastic cuvettes, 1x1x4 cm, with relevant solutions were prepared immediately before the measurements. The cuvettes were covered by parafilm to minimize the air exposure of the samples, which would accelerate the sample degradation and decrease the pH value. The fluorescence spectra of DOX with a concentration of c = 2 × 10^−3^ mg/mL (36 μM) in water were performed in a pH range of 5.5–10.0. To determine the binding constants, the samples containing 0.5 mL of DOX with a concentration of c = 2.9 × 10^−4^ mg/mL (5 μM) were mixed by adding 0.5 mL of G4.0 PAMAM solution to obtain a final dendrimer concentration in the range of c = 7.1 × 10^−3^–0.1 mg/mL (5–75 μM). The binding constant Ka was calculated using the following equation [65]:

Log [(F_0_/F)/F] = Log*K_a_* + n log[*Q*] (6)

A plot of log[(F0/F)/F] versus log[Q] gives a straight line. From the slope of the linear curve, we can obtain the value of the binding constant *Ka*.

The G4.0-DOX fluorescence measurements were carried out for the post-dialysis complexes with an initial G4.0-DOX molar ratio of 1:12 and 1:24 and an initial pH of 9.5. The exact methodology for preparing the complexes is described in our previous work [29]. Briefly, sufficient DOX at pH = 9.5 was added to the dendrimer at a constant concentration of 0.25 mg/mL (17.6 μM) and pH = 9.5 to give a molar ratio of the starting complexes of 1:12 and 1:24. The complexes were mixed for 24 h and dialyzed using Slide-A-Lyzer™ Dialysis Cassettes (MWCO 10.0 kDa), Thermo Fisher (Waltham, MA, USA) for another 24 h to remove the drug molecules not bound to the dendrimer. The final pH of the post-dialysis complexes stabilized at pH = 7.5. All the fluorescence spectra were measured at an excitation of λ_ex_ = 482 nm and the emission was recorded in range λ_em_ = 490–700 nm.

### 4.9. Isothermal Calorimetry (ITC) Measurements

The ITC experiments were carried out using a Nano ITC calorimeter (TA Instruments) with a standard volume of 1.0 mL at 25 °C. All the solutions were prepared in deionized water (>18 Ω) and PBS buffer solution. All the solutions used to fill the cell and the syringe were degassed before analysis. The reference cell was filled with deionized water. The G4.0 PAMAM dendrimer was an analyte (0.0176 mM) in the cell, and DOX (2.12 mM) was a titrant in the syringe. Each time, freshly prepared titrant solution was taken up in a 250 μL injection syringe and titrated into fresh G4.0 PAMAM solution. A total number of 25 (10 μL) or 50 injections (5 μL) were added after the calorimeter finalized the primary equilibration. The intervals between the injections were 200 s and 300 s, respectively, leaving 200 s at the beginning of the experiment without injection. The stirring rate was set at 300 rpm. The control experiments to determine the heat of the dilution of DOX were performed by injecting the same concentration of DOX into the buffer. The calorimeter was operated using the Nano ITC Run software, and all the data obtained were analyzed using the NanoAnalyze v. 3.1.2 program provided by the manufacturer. An “independent” and “multiple sites model” were used to evaluate the results. At least three independent measurements were collected, and the ITC data and reproducible data were employed. For thermodynamic parameters calculation, a total number of 25 (10 μL) injections were used.

### 4.10. Release Studies

The release tests were performed for the G4.0-DOX complex at a molar ratio of 1:6 and pH = 9.5. The complex was prepared in accordance with a previously developed methodology [29]. The concentration of the components in the final complex was c_G4.0_ = 250 μg/mL, c_DOX_ = 22 μg/mL (EE = 37.93%). The pH of the complex stabilized at pH = 7.5. An aqueous solution of DOX with c = 22 μg/mL and pH = 7.5 was prepared as a reference solution. The prepared G4.0-DOX complex and the reference DOX solution were placed in dialysis membranes (Thermo Scientific™ Slide-A-Lyzer™ MINI Dialysis Devices, MWCO 3.5 kDa). The dialysis bag was submerged in 45 cm^3^ of Dulbecco’s PBS buffer at pH 7.4, and was incubated at 25 °C for 96 h. The released DOX in the incubation buffer was analyzed at predetermined time intervals using a Thermo Scientific Evolution 201 UV-vis spectrophotometer by measuring the spectrum in the wavelength range of λ = 190–800 nm. The concentration of the released DOX was determined using the previously determined extinction coefficient ε_DOX pH 7.5_ = 9610 M^−1^ cm^−1^ at λ_max_ = 480 nm [29]. The kinetic parameters were determined from the curve fitting and calculated using the following equation:

*y = a(1 − e^−bx^)* (7)

where

*y—DOX released [%]*;

*x—time [h]*;

*a—*maximal amount of released DOX (A_max_) [%];

*b—*release rate constant (k_1_) [h^−1^].

### 4.11. Cell Culture and Cytotoxicity of the Systems against Malignant Cells

For the study, the following cell lines were used: colon cancer cell lines (HT-26, LoVo, and LoVoDX), lung cancer cell lines (NCI-H23 and H69AR), breast cancer cell lines (MCF-7 and MCF-7/DX), skin cancer cell line A375, and immortalized keratinocytes (HaCaT) as a model of the normal cell line. The cells were maintained at 37 °C and 5% CO_2_, the colon cancer cells were maintained in a Ham’s/F12 medium, and the other lines in a Dulbecco’s Modified Eagle’s Medium (Sigma-Aldrich) supplemented with 10% fetal bovine serum (fetal bovine serum, Heat-Inactivated, non-USA origin, sterile-filtered, Sigma-Aldrich) and antibiotics: 100 IU/mL penicillin and 0.1 mg/mL streptomycin (Gibco, Gaithersburg, MD, USA). For the experiments, the cells were removed from culture flasks (Sarstedt, Warsaw, Poland) with a 0.25% trypsin-EDTA solution (IITD, Wroclaw, Poland), and suspended in a 96-well plate (Sarstedt) at a cell density 2 × 10^4^/mL. Then, the cells were maintained for 24 h in a CO_2_ incubator for cell attachment. The next day, the plate medium was removed and 200 µL of DOX/dendrimers/conjugates diluted with a culture medium was added to the appropriate wells for 24 and 72 h. The final concentration of DOX conjugated with the G4PAMAM dendrimers or unconjugated was 1, 2, 4, and 8 µM. Blank vehicle controls as the equivalents of these concentrations were also examined after the incubation cell viability was examined with the MTT assay, as previously described in [66].

The absorbance was measured at 560 nm with the GloMax^®^ Discover Microplate Reader (Promega, Beijing, China). The results presented on the plots were blank-subtracted and normalized to the non-treated control. The cell viability values were represented as a percentage of the untreated control cells (100%). The experiments were performed in triplicates. One-way ANOVA followed by Dunnett’s multiple comparisons test was performed with the GraphPad Prism software, version 7.0. (GraphPad Software, San Diego, CA, USA). Dunnett’s multiple comparisons test was chosen as it allows for comparing multiple treatment groups against a single control group, which aligns with our experimental design. Differences between the treated samples and control cells with *p ≤* 0.05 were taken to be statistically significant.

### 4.12. Intracellular Distribution of Doxorubicin by CLSM Study

Two cell lines were used to distribute free and encapsulated DOX intracellularly: skin cancer cells—A375 and normal keratinocytes—HaCaT. The cells were trypsinized and placed on microscopic cover round glasses (Φ18 mm, Carl Roth GmbH, Karlsruhe, Germany) in a 6-well plate (Equimed, Poland). The cells were seeded on a 96-well plate for intravital imaging. In both cases, the cells were left for 24 h to adhere and subsequently exposed to free DOX, DOX in nanocarriers, and empty nanocarriers for 24 h or 72 h. After that, the cells were fixed by exposing them for 10 min to 4% paraformaldehyde (Polysciences, Inc., Bergstrasse, Germany) and washed in PBS (IITD, Wroclaw, Poland). FluorshieldTM with DAPI (4,6-diamidino-2-phenylindole) (Merck, Sigma-Aldrich, Poznan, Poland) was used to counterstain the nuclei and to mount the cells, and was excited by the 405 nm-excitation channel. Intravital imaging was performed after 24 h. The samples were studied on the MICA Microhub confocal microscopy (Leica Microsystems, CellService, Poznan, Poland).

## 5. Conclusions

To sum up, our studies illustrate that doxorubicin, which occurs in a protonated form in a physiological environment, becomes deprotonated at a higher pH. These are ideal conditions for forming a complex between the positively charged G4.0 PAMAM dendrimers and negatively charged doxorubicin. It has been shown that at low concentrations of DOX in the absence of self-association, the drug forms an effective complex with the G4.0 PAMAM dendrimer. The conditions for the complex formation determine the drug’s affinity, which depends on the pH and the degree of the ionization of the components. The dendrimer endothermically binds doxorubicin at two binding sites—hydrophobic spaces and on charged surface groups. This is possible due to the properties of the DOX molecule and the presence of a hydrophobic anthraquinone in its structure, as well as the functional groups susceptible to ionization and electrostatic interactions. This is important in the application context since the localization of the drug in the carrier structure determines both the release kinetics, which in the case of the G4.0-DOX nanosystem is pH-dependent, and the in vitro activity. The observed increase in the IC50 values with higher DOX loading ratios in the PAMAM-DOX complexes is attributable to the complex interplay between drug release dynamics, dendrimer saturation, steric hindrance, and aggregation effects. These factors collectively influence the bioavailability and cytotoxic potential of DOX when complexed with PAMAM dendrimers. Moreover, the observed lower toxicity of the DOX-dendrimer complex after 24 h highlights the benefits of controlled drug delivery systems in modulating drug release and improving selectivity. In conclusion, our results support the potential of dendrimer-based complexes to enhance the therapeutic window of DOX, providing a strategic advantage in cancer treatment by reducing toxicity to normal cells while maintaining efficacy against cancer cells.

## Figures and Tables

**Table 1:** The number of dendrimer particles (N_G4.0_) adsorbed on the Au sensor and doxorubicin (N_DOX_) on the dendrimer surface in the MP-SPR method depending on the pH of DOX (pH_G4.0_ = 10.0, c_G4.0_ = 0.25 mg/mL, c_DOX_ = 0.06 mg/mL, H_2_O).

pH_DOX_	N_G4.0_ (×10^13^)	N_DOX_ (×10^13^)	N_DOX_/1 G4.0 Particle
**9.0**	8.1	6.2	0.8
**9.5**	8.7	24.9	2.9
**10.0**	9.2	156.0	16.9

**Table 2:** Thermodynamic parameters obtained for the G4.0/DOX system in the ITC experiments. Data for the titration of 2.12 mM DOX into 0.0176 mM G4.0 in PBS buffer, pH 7.4. T = 25 °C.

Binding Site	K_a_ (M^−1^)	n	ΔH (kJ/mol)	K_d_ (M)	ΔS (J/mol K)	ΔG (J/mol)
1	1.215 × 10^5^ ± 0.55	6.18 ± 0.30	9.068 ± 0.26	8.232 × 10^−6^	1.278 × 10^2^	−2.902 × 10^4^
2	1.065 × 10^2^ ± 0.51	17.05 ± 4.85	198.9 ± 57	9.390 × 10^−3^	7.058 × 10^2^	−1.143 × 10^4^

**Table 3:** The half maximal inhibitory concentration (IC50) values and the selectivity index (SI) values of doxorubicin alone (DOX) compared to the G4.0-DOX complexes in 3 cell lines: normal keratinocytes (HaCaT), malignant melanoma (A375), and lung cancer (NCl-H23) following a 72-h incubation with the compounds.

Cell Line	HaCaT		A375		NCl-H23	
**Compound IC50**						
	IC50 [μg/mL]	IC50 [μM]	IC50 [μg/mL]	IC50 [μM]	IC50 [μg/mL]	IC50 [μM]
**DOX**	0.37	0.64	1.14	2.41	0.55	0.95
**G4.0-DOX 1:6**	0.64	1.10	4.22	7.28	3.64	6.28
**G4.0-DOX 1:12**	0.58	1.00	0.73	1.26	0.72	1.24
**G4.0-DOX 1:24**	0.81	1.40	-	-	2.28	3.93
**G4.0-DOX 1:50**	0.82	1.41	3.57	6.16	1.89	3.26
**Compound SI**						
**DOX**	-		0.32		0.67	
**G4.0-DOX 1:6**	-		0.15		0.18	
**G4.0-DOX 1:12**	-		0.79		0.81	
**G4.0-DOX 1:24**	-		-		0.38	
**G4.0-DOX 1:50**	-		0.23		0.43	

**Table 4:** Maximum percentage of the released mass (A_max_) and release rate constant (k_1_) of DOX from the complex compared to free drug into the PBS buffer at pH = 7.4.

	pH	A_max_ [%]	k_1_ [h^−1^]	R^2^
**DOX**	4.0	99.2 ± 1.7	0.062 ± 0.003	0.99
	7.5	91.6 ± 2.6	0.070 ± 0.008	0.97
**G4.0-DOX**	4.0	97.9 ± 2.1	0.082 ± 0.007	0.98
	7.5	73.3 ± 2.4	0.091 ± 0.012	0.95

## Data Availability

All data generated or analyzed during this study are included in this published article.

## Supplementary Materials

The following supporting information can be downloaded at https://www.mdpi.com/article/10.3390/ijms25137201/s1.
