# Supplementary material for: Dendrimer Platforms for Targeted Doxorubicin Delivery—Physicochemical Properties in Context of Biological Responses
Source: Int J Mol Sci. 2024 Jun 29;25(13):7201. doi: 10.3390/ijms25137201 (PMC11241532; doi:10.3390/ijms25137201)
Supplement: Supplementary file 1 [file ijms-25-07201-s001.zip › ijms-3015418-supplementary.pdf]

Supplementary Materials

Dendrimer Platforms for Targeted Doxorubicin Delivery – Physicochemical Properties in Context to Biological Responses

Magdalena Szota [1], Urszula Szwedowicz [2], Nina Rembiałkowska [2], Anna Janicka-Kłos [3], Daniel Doveiko [4], Yu Chen [4], Julita Kulbacka [2] and Barbara Jachimska [1,*]

[1] Jerzy Haber Institute of Catalysis and Surface Chemistry Polish Academy of Sciences, 30-239 Cracow, Poland
[2] Department of Molecular and Cellular Biology, Faculty of Pharmacy, Wroclaw Medical University, 50-367 Wroclaw, Poland
[3] Department of Basic Chemistry, Wroclaw Medical University, 50-367 Wroclaw, Poland
[4] Department of Physics, University of Strathclyde, G4 0NG Glasgow, UK

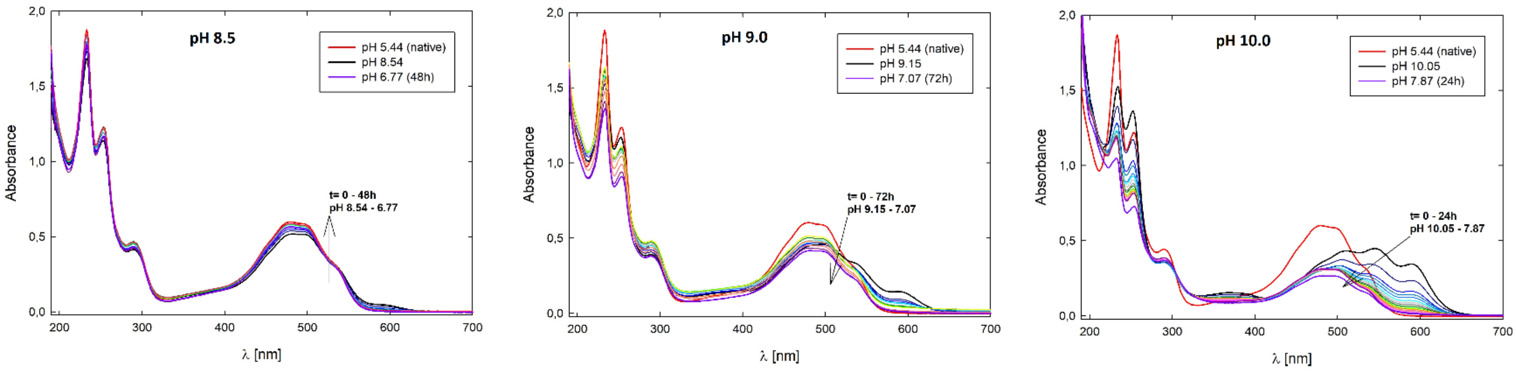

Figure S1. UV-Vis spectra of doxorubicin versus time and pH (c= 50 μg/mL, H_2O).

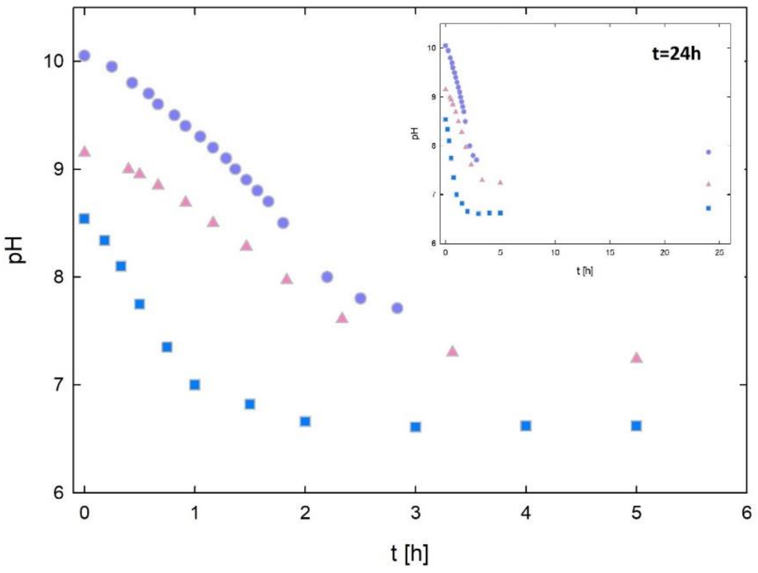

Figure S2. Decrease in pH of doxorubicin solution over time as a function of pH: pH= 10 (purple curve); pH= 9.0 (pink curve) and pH= 8.5 (blue curve).

Table S1. Hydrodynamic diameter (d_H), polydispersity index (PDI) and zeta potential (ζ) of DOX aggregates in water solution (c=0.5 mg/mL).

pH	d_H [nm]	PDI	ζ [mV]
**5.2**	440.8 ± 64.6	0.89 ± 0.09	40.0
**7.5**	426.4 ± 76.1	0.59 ± 0.05	39.7
**8.5**	574.5 ± 62.5	0.44 ± 0.03	36.9
**9.0**	> 10^3	0.36 ± 0.10	30.7
**9.5**	> 10^3	0.47 ± 0.16	18.3
**10.0**	> 10^3	0.47 ± 0.15	-0.1

The particle size distribution shows the formation of DOX aggregates of 440.8 ± 64.6 nm at pH=5.22. A similar aggregate size is observed at pH 7.5 and 8.5, with 426.4 ± 76.1 and 574.5 ± 62.5 nm, respectively. The PDI values indicate the polydisperse nature of the system. Above pH=9.0, aggregation strongly increases to values above 1,000 nm, which may be favored by the instability of the aggregate system.

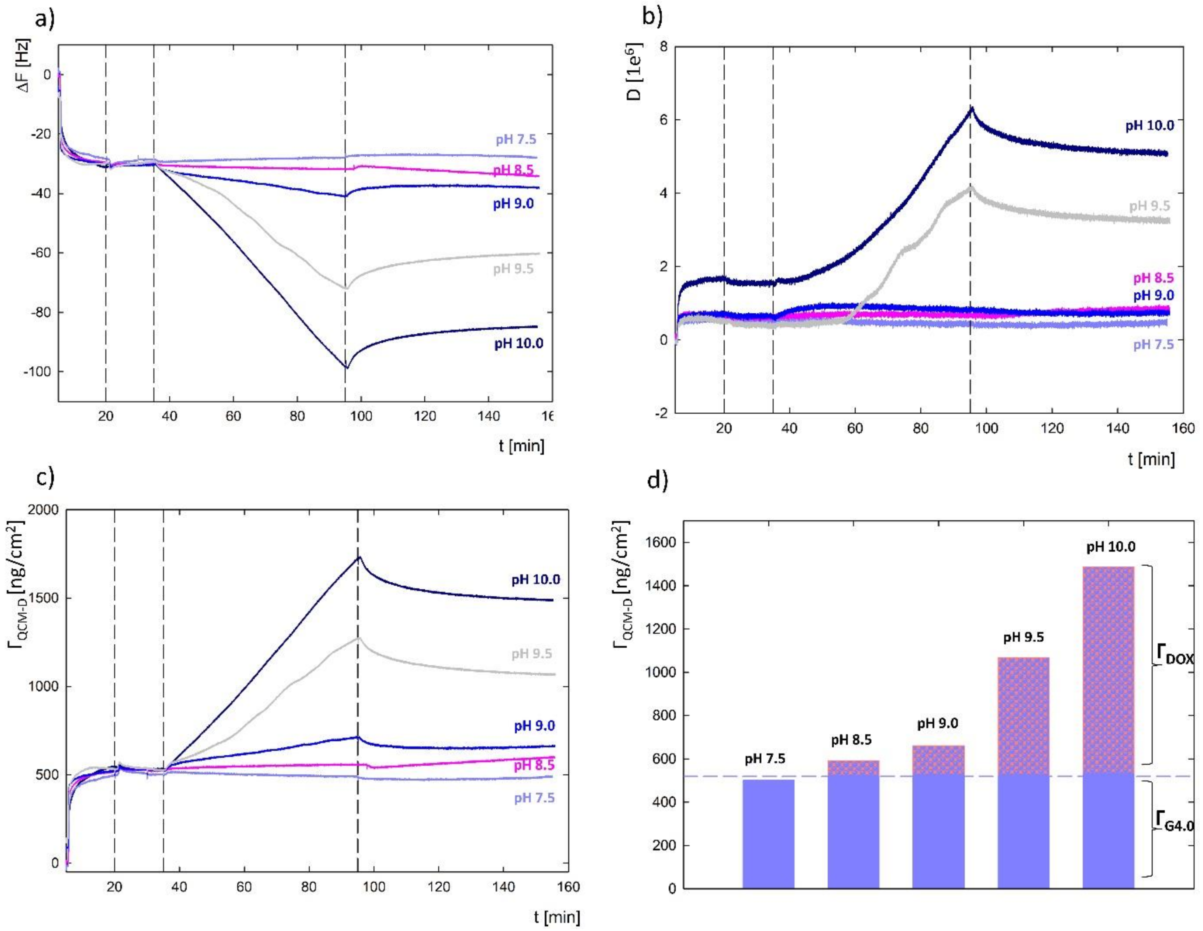

Figure S3. QCM-D results for G4.0-DOX bilayers formation at pH range of DOX solution from 7.5 – 10.0 (G4.0/ DOX molar ratio 1:6, $c_{G4.0}$ = 17.6μM, $pH_{G4.0}$ = 10.0, water); (a) Time dependence of the resonance frequency (ΔF) of the QCM-D sensor's vibrations for G4.0 PAMAM/DOX bilayers; (b) Time dependence of Dissipation of adsorbed bilayers (D); (c) Time dependence of the mass of adsorbed bilayers (Γ_{QCM-D}) on the gold surface; (d) comparison of adsorbed mass of bilayers after rinsing.

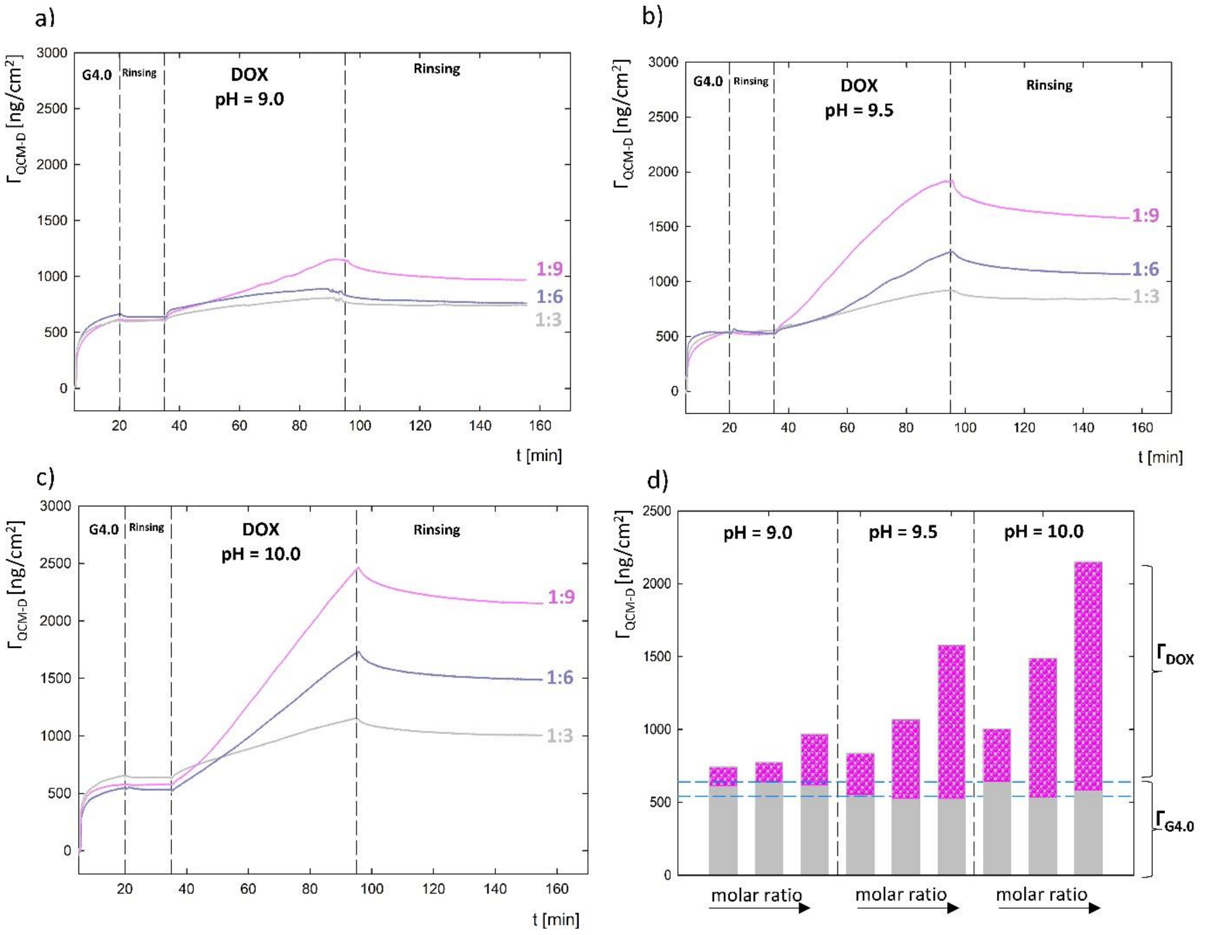

Figure S4. QCM-D results for G4.0-DOX bilayers formation at molar ratio range from 1:3 – 1:9 at different pH ($c_{G4.0}$ = 17.6μM, $pH_{G4.0}$ = 10.0, water); (a) Time dependence of the mass of adsorbed bilayers (Γ_{QCM-D}) at DOX pH = 9.0; (b) Time dependence of the mass of adsorbed bilayers (Γ_{QCM-D}) at DOX pH = 9.5; (c) Time dependence of the mass of adsorbed bilayers (Γ_{QCM-D}) at DOX pH = 10.0; (d) comparison of adsorbed mass of bilayers after rinsing.

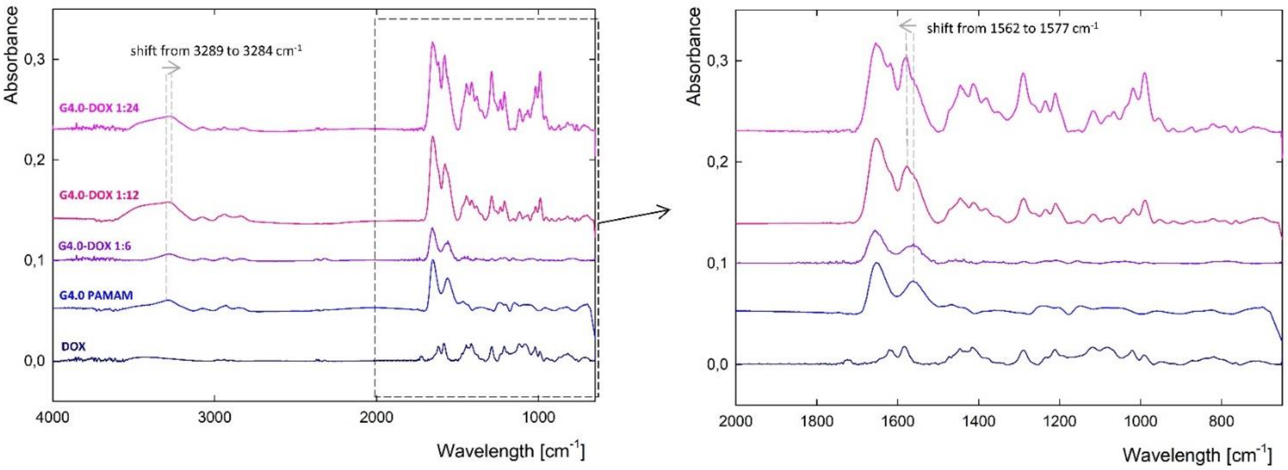

Figure S5. FTIR spectra of G4.0-DOX complexes compared to free DOX and G4.0 PAMAM (from top to bottom) ($c_{G4.0}$ = 0.25mg/mL, c_{DOX} = 0.24 mg/mL, H_2O, pH = 9.5).

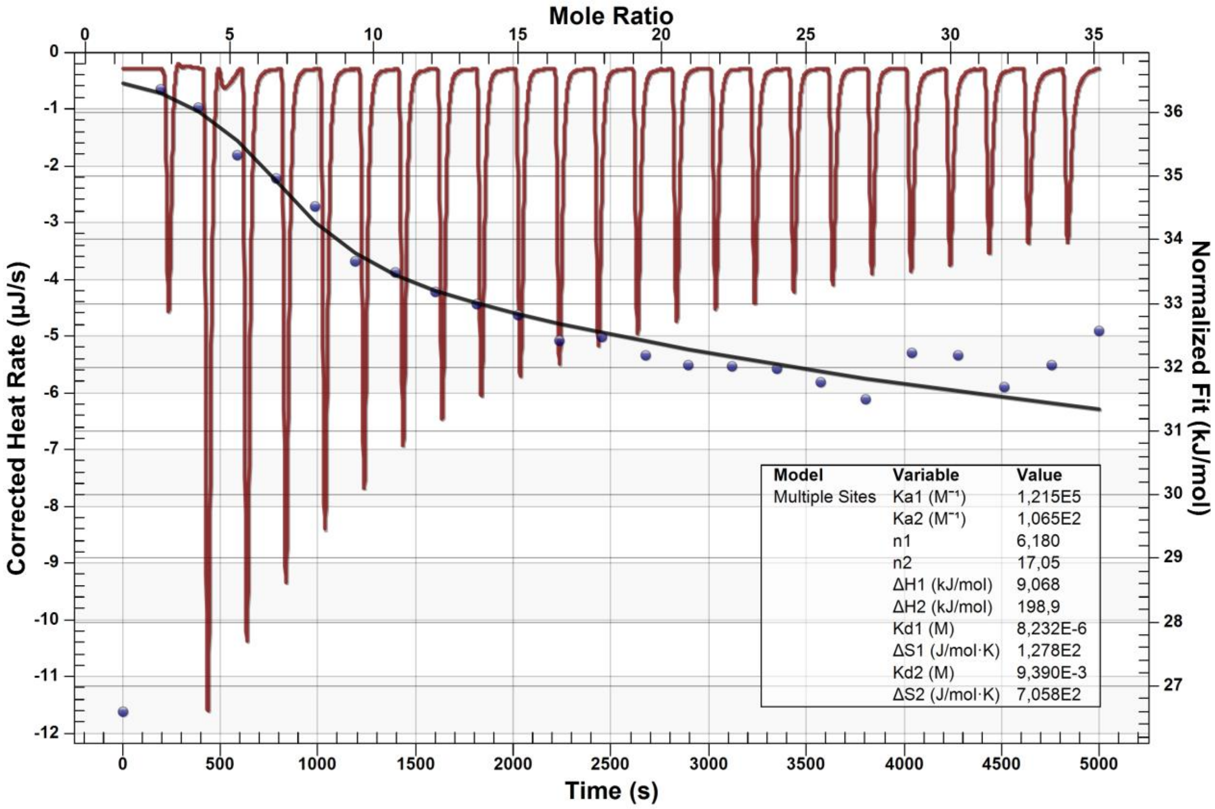

Figure S6. The best-fit ITC data for the titration of 2.12 mM DOX into 0.0176 mM G4.0 PAMAM in PBS buffer, pH 7.4 and T= 25 °C.

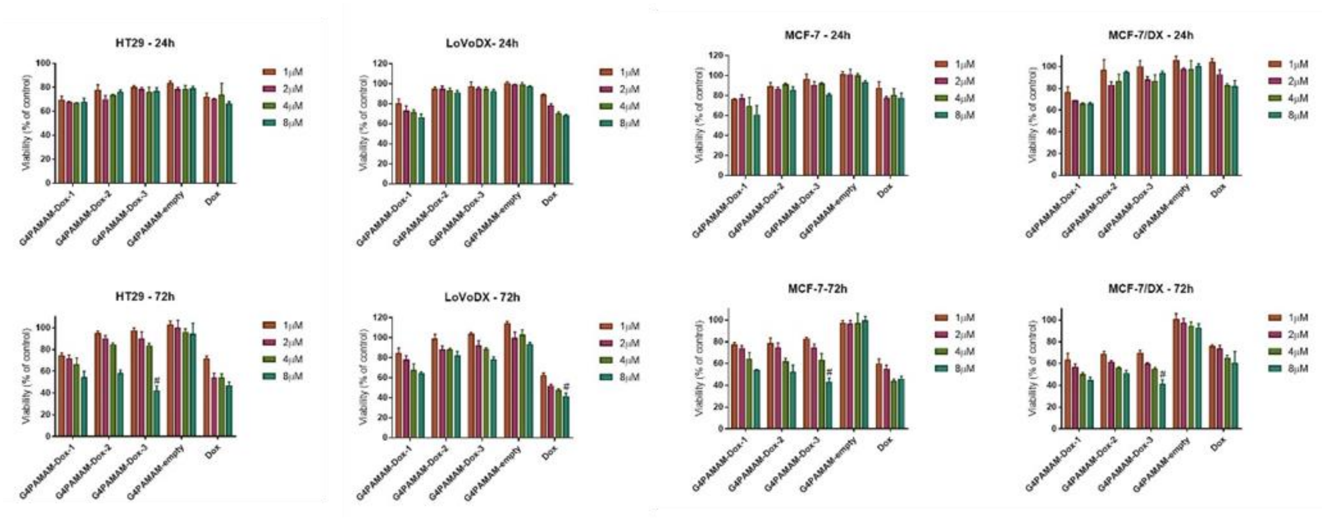

Figure S7. The cytotoxic effect of G4.0 PAMAM nanocarriers against human cancer (HT29, MCF-7, MCF-7/DX) cell linse, evaluated by MTT assay after 24 and 72h. # p≤0.05.
